# Client-centered counseling improves client satisfaction with family planning visits: evidence from Irbid, Jordan

**DOI:** 10.9745/GHSP-D-12-00051

**Published:** 2013-07-26

**Authors:** Sarah Kamhawi, Carol Underwood, Huda Murad, Bushra Jabre

**Affiliations:** a Jordan Health Communication Partnership, Amman, Jordan; b Johns Hopkins Bloomberg School of Public Health, Baltimore, MD, USA

## Abstract

**Background:**

High levels of unmet need for family planning and high contraceptive discontinuation rates persist in Jordan, prompting the Jordan Health Communication Partnership (JHCP) to initiate a client-centered family planning service program called “Consult and Choose” (CC), together with community-based activities to encourage women with unmet need to visit health centers.

**Methods:**

We held exit interviews with 461 family planning clients between November–December 2011 to assess, from the clients' perspective, whether trained providers followed the CC protocol and used the CC tools, as well as to measure client satisfaction. We also tracked referral card information from community-based activities to health centers and examined service statistics to explore trends in family planning use.

**Results:**

On average, clients reported that providers performed 5.6 of the 7 steps outlined in the CC protocol. Nearly 83% of respondents were very satisfied with their clinic visits. Logistic regression analysis found that the odds of being “very satisfied” increases by 20% with each additional counseling protocol step performed and by 70% with each increase in the number of CC materials used. Between June 2011 and August 2012, 14,490 referral cards from community-based activities were collected in health centers, 59% of which were for family planning services. Service statistic trends indicate an increase in the number of new family planning users and in couple-years of protection after starting the CC program.

**Conclusions:**

Implementation of the CC program at health centers nationally, in tandem with community-based interventions, could play a key role in attaining Jordan's goal of reducing its total fertility rate to 2.1 by 2030. Although this initiative would likely be replicated most readily in other middle-income countries, lower-resource countries could also adapt the tested CC approach.

## Abstract (precis)

In Irbid, Jordan, a combination of community outreach, using home visits, plays, women's groups, and religious leaders, and improved client-provider counseling based on the “Consult and Choose” approach increased family planning demand and client satisfaction. Service statistic trends suggest increased contraceptive use.

## INTRODUCTION

Providers play a key role in a woman's decision to use contraception, as well as in which method she selects and her adherence to the chosen method.^1^-^2^ One key element of Bruce's framework for assessing the quality of family planning services from the client's perspective is interpersonal relations between client and provider.^2^ A study in Egypt found that client satisfaction was 3 times higher with client- versus provider-centered counseling, and continuation rates were higher at 7 months after the visit.^3^ Another study found that providing information to clients about the hormonal IUD prior to inserting it was significantly associated with high client satisfaction.^4^ Structured counseling greatly influences a client's choice of method^5^ and is associated with lower discontinuation rates, particularly with the injectable *Depo-Provera®*, known to have a high discontinuation rate.^6^ Additionally, a systematic review of the literature on the acceptability of the intrauterine device (IUD) demonstrated that women who received structured counseling and referrals from community workers had higher uptake of the IUD compared with women in control groups who received standard care.^7^

Screening tools and client decision aids also can play a key role in enhancing the client's experience.^8^ The use of the tools alone is limiting, however. For example, 2 independent studies in Nicaragua revealed that use of the World Health Organization's (WHO's) “Decision-Making Tool for Family Planning Clients and Providers” was not significantly associated with improved continuation rates,^9^ even though clients were more satisfied with the counseling they had received. ^9^-^10^

These findings are relevant to Jordan, where efforts to increase access to and use of modern contraception have been ongoing for several decades. The contraceptive prevalence rate (CPR) among married women ages 15–49 years is 59%, according to the 2009 Jordan Population and Family Health Survey (JPFHS).^11^ Most married women report modern method use (42%), but 17% rely on traditional methods.

A 2006 study conducted in Ministry of Health (MOH) national health centers demonstrated that 16% of married women had an unmet need for family planning—that is, they wanted to avoid pregnancy altogether or for at least 2 years but were not using a method.^12^ This unmet need was significantly and negatively associated with women's educational attainment. High levels of method discontinuation (45% of married women discontinue their method within the first 12 months of use)^11^ contribute to unmet need in Jordan. The most cited reasons for discontinuation, besides wanting to become pregnant, are method failure (17%), wanting a more effective method (13%), and side effects (12%).^11^

Unmet need among married Jordanian women may be associated with the quality of family planning counseling they receive. A national qualitative study examining the knowledge, attitudes, and practices of family planning service providers from the MOH revealed that providers' personal beliefs greatly influence their counseling. Most providers were not convinced by the small family size concept, instead emphasizing the importance of a large family for one's social status and the preference for a male child.^13^ The providers' religious beliefs influenced the type of method they recommended, and many providers indicated that they recommend short-term methods to married women with low parity and would not suggest, for example, the IUD for such women for fear that it would affect their fertility.^13^

Provider biases can restrict clients' choice of contraceptive method.

Moreover, most providers reported that they did not provide family planning counseling due to time constraints. When they did counsel clients about family planning, it was often rushed, conducted in an open setting, and incomplete in that most physicians failed to discuss the side effects associated with the client's chosen method.^13^ Surprisingly, most service providers acknowledged that improper counseling results in discontinuation and an increase in unmet need.^13^

In response to high discontinuation and unmet need rates, the Jordan Health Communication Partnership (JHCP) sought to enhance the quality of service provision by strengthening providers' counseling skills and approach through the “Consult and Choose (CC)” program. The program was informed by the understanding that behavior change is a function of beliefs, attitudes, and intentions^14^-^15^ that are influenced by external factors, such as perceptions about the social normative practices of peers, socioeconomic circumstances, and the broader health care system.

In the context of the decision to use contraception, on the individual and social-network levels, couples must be convinced that a small family is beneficial, feel the need to space their children, be free of familial pressure to have large families, and be sufficiently informed to distinguish between rumors and facts. In addition, each woman has to feel that she is capable of delaying or preventing her pregnancy and that modern contraceptives are safe. At the same time, she needs to have ready access to contraceptive products and services, including professional counseling (systemic factors).

The guiding hypothesis of the CC approach was that the more comprehensive the communication interventions, the more positive the clinic experience. This positive experience would then be associated, in turn, with increased adoption and sustained use of modern contraceptive methods.

In this article, we describe the CC program design and implementation; present findings on whether health care providers' use of the CC materials is associated with clients' satisfaction; and discuss implications of the CC experience for other middle-income countries. (Testing whether satisfaction with clinic visits is associated with the adoption and sustained use of modern methods was beyond the scope of our evaluation.)

## THE CONSULT and CHOOSE INTERVENTION

The CC program is part of the larger Irbid Initiative that takes into account several (but not all) personal and systemic factors that influence family planning use and is based on a “push-pull” approach. Community-level interventions encourage women to go the health center while advocating for husbands to support their wives' decisions (the push factor). Concurrently, the program equips health service providers with the necessary tools and skills to interact effectively with their clients so they can help clients make appropriate choices (the pull factor).

The overall objectives of the CC program are to:

- Increase the percentage of women using modern contraception
- Create a sense of belonging between modern contraceptive users
- Empower potential and current contraceptive users by connecting them with health centers as resources for accurate, credible, trustworthy family planning-related information and quality services (based on *Hayati Ahla* [My Life is Better] messaging, the national family planning campaign)
- Introduce counseling as a partnership between the client and service provider for informed decision-making
- Overcome service providers' biases by fostering professionalism and positive attitudes about modern contraceptives, pregnancy spacing, and small family size

### The Push Factor

The Irbid Initiative gave technical and financial assistance to 9 Community Health Committees in Irbid governorates to implement community-based interventions, such as plays, debates, awareness sessions, and home visits. Another key community-based activity was Arab Women Speak Out^TM^ (AWSO^TM^), which brought together groups of about 20 women of reproductive age to discuss issues related to marriage and family health, including contraceptive use and the need to go to the health center. Guided by a facilitator and inspired by stories of role models, the discussions revolved around issues and beliefs related to family planning, thus bringing these traditionally private topics to the public sphere. AWSO^TM^ addressed predisposing factors of knowledge, attitude, beliefs, and values as well as enabling factors, such as the supporting environment and community. Participants received flash cards containing summaries of key topics so that they could then disseminate the messages to others within their community.

Arab Women Speak Out^TM^ brings together women from the community to discuss marriage and family health issues.

In addition, all 776 religious leaders in Irbid attended a 3-day training program that focused on family health and highlighted Islam's approval of family planning and modern contraceptives. Through sermons and religious lessons in mosques, religious leaders conveyed their approval of family planning, thereby overriding the notion held by a small, but sometimes vocal, group that Islamic precepts prohibit modern contraception.

Finally, all health promoters, one from each health center in Irbid, were trained to use JHCP's materials so that they could play a more effective role in promoting family planning in a given health center's catchment area.

The project hypothesized that the push factor would get women to the clinics.

### The Pull Factor

The CC program blends the approaches recommended by the literature on family planning counseling into a single package for use by health care providers trained to use the following nationally distributed materials (see http://www.k4health.org/toolkits/jhcp/family-planning-service-provision-toolkit):

- The WHO's Medical Eligibility Criteria Wheel for Contraceptive Use and *Family Planning: A Global Handbook for Family Planning Providers* with the accompanying Family Planning Wall Chart (published jointly by the Johns Hopkins Bloomberg School of Public Health Center for Communication Programs and WHO), which provide detailed medical information relating to contraindications and eligibility for each contraceptive method
- Service Provider Cue Cards for 8 contraceptive methods, which include a picture of the method, description of its effectiveness, major advantages and side effects, how to manage side effects, and how to use the method
- Client Cue Cards for every modern method that the client takes home, which includes a picture and the name of the client's chosen method, when to return, how to deal with side effects, grace period if a dose is missed, pointers for a dialogue with one's husband, and religious messages
- Posters displaying the clinic's promise to provide high-quality services to the client

Providers in Irbid had previously received extensive 5-day training in family planning counseling techniques and approaches by another agency. In 2010–2011, the CC program trained all Irbid family planning service providers during a half-day session to orient them on the CC tools and to reinforce proper counseling. Using a film produced by JHCP that models proper counseling (using the Rapport Building, Exploration, Decision-Making, Implementation counseling framework^16^) and use of the CC materials as a starting point, participants had an open and frank discussion about their own counseling practices.

The CC tools help limit the negative effects of any provider biases by providing step-by-step guidelines that adhere to internationally recognized standards. Proper counseling sessions using the CC approach typically last from 5 to 15 minutes, depending on whether clients are new or returning. After the training session, participants received a branded *Hayati Ahla* lab coat (although a few female providers preferred to continue wearing the traditional long blue lab coat) and pin, and they gave an oath to adhere to proper CC counseling.

JHCP, in collaboration with the MOH, also produced a 15-minute film for clients titled *Hayati Ahla*. The video was shown in maternal and child health (MCH) waiting areas in 6 Irbid-based centers and included information about JHCP and all its government and non-governmental partners, campaign ads by JHCP and its partners, and information about the MCH center. It also included short testimonials about postabortion family planning and family planning advice and guidance from influential professionals from the health sector.

The project hypothesized that the “pull factor” activities would contribute to clients' overall satisfaction. The guiding hypothesis of the CC approach was that overall satisfaction would be high in the sampled centers, that satisfaction would be correlated with providers' use of CC materials and adherence to counseling guidelines, and that beneficiaries who received CC materials would read them and find them useful. In addition, we hypothesized that the *Hayati Ahla* film in waiting areas would have high rates of viewership and that viewers would recall messages that correspond to the film's content.

## METHODOLOGY

We conducted exit interviews with family planning clients at MCH centers to evaluate, from their perspective, providers' adherence to the CC counseling protocol and use of CC materials, as well as to assess client satisfaction with the counseling experience. We also tracked referrals to health centers from community-based activities and used service statistics from the MCH centers to explore contraceptive use trends since starting the CC program.

The Jordanian MOH reviewed and approved the evaluation protocol and the Institutional Review Board at the Johns Hopkins Bloomberg School of Public Health provided ethical approval for the evaluation.

### Exit Interviews at MCH Centers

#### Sample Selection

We relied on a post-test only single sample design because we distributed CC materials to MCH centers throughout Jordan, thus eliminating the option of a control sample.

We stratified the sample by district and selected 20% of the 100 MCH centers in the 9 districts in Irbid. We based selection on client flow, so we included centers with the highest number of family planning beneficiaries in the sample. Centers that showed the *Hayati Ahla* video were automatically included. All selected centers provided IUD services.

Women were randomly selected for screening prior to inclusion in the evaluation. Women ages 15–49 and who were engaged or married at the time were eligible for inclusion. Women who met these criteria were then asked to specify why they were visiting the MCH center. Of the randomly selected women, those who were visiting the center for any family planning service were included in the evaluation. Family planning services included obtaining a method, receiving counseling, and/or following up or consulting about a currently used method.

#### Questionnaire

The interviewer-administered questionnaire included 28 questions about demographic characteristics, the family planning counseling or service experience, and satisfaction with the health center. We also asked women who were visiting the 6 health centers that displayed the *Hayati Ahla* video an additional 11 questions about the video. No identifiers were collected.

#### Data Collection, Entry, and Analysis

All interviews took place after the client exited from her session with the health care provider. When referring to CC materials used during counseling or consultations, the interviewers showed the respondents the CC materials of interest. We assessed providers' use of CC materials, including the medical eligibility criteria wheel, provider and client cue cards, and the *Hyati Ahla* lab coat, based on the client's recollection of the provider using these materials. Internal consistency of the resulting scale was satisfactory (Cronbach's alpha = 0.67). We also assessed providers' adherence to the CC family planning counseling protocol based on the client's recollection of the provider discussing 7 key issues before choosing a method, such as ideal family size and advantages and disadvantages of available methods, as well as how to manage side effects of the client's chosen method (Cronbach's alpha = 0.86).

We assessed client satisfaction using a 4-point scale (very satisfied, somewhat satisfied, dissatisfied, or very dissatisfied). We instructed interviewers to select a pre-coded response of “unsure or no opinion” if the client expressed uncertainty or neutrality. We then transformed the scale into a dichotomous variable, where a positive response was recorded when the beneficiary was “very satisfied,” for the data revealed that most beneficiaries where somewhat or very satisfied.

The research team used CSPro 4.0, a public-domain software package for entering, editing, and tabulating survey data. The data set was cleaned and edited for inconsistencies. Missing data were not statistically included. We used STATA version 11 statistical software to complete the data analysis.

Chi-square testing was used to determine statistical significance when comparing proportions. Stepwise multivariate logistic regression analysis was conducted to explore the relationship between the odds of being very satisfied with the odds of not being very satisfied and the adherence to proper family planning counseling and use of CC materials, while accounting for age and educational attainment.

### Service Statistics

To measure the mobilization effect of the community-based interventions, women who had participated in AWSO^TM^ or who had attended community-based activities or sermons and who had unmet health needs were given a “referral card” to take to an MCH center when they sought services. Referral card information was recorded at the center and sent to the MOH's quality control department on a monthly basis for analysis, which was then fed back to the field.

We also used 2 measures from service statistics collected from MCH centers in Irbid over the past 4 years to examine trends in family planning use:

- Number of new family planning clients: A woman who visits the MCH center for any family planning service for the first time is entered into the MCH information system as a “new client.” Data were summed from each of the 9 districts in the Irbid governorate as a surrogate measure for family planning demand (being a “new client” does not necessarily reflect uptake of a contraceptive method).
- Couple-years of protection (CYP): The CYP for the specified time periods was calculated by the Irbid health directorate and validated at the central level of the MOH.

## RESULTS

### Quantitative Findings From Exit Interviews

#### Recruitment and Characteristics of Respondents

We completed 461 client interviews between November 23, 2011 and December 5, 2011. About three-quarters of the women (n = 352) received family planning counseling during that day's visit (Table 1). A total of 277 were there to follow up or consult about a method they were already using, 234 of whom had been counseled on their method of choice at the health center during a previous visit. Because the CC materials were primarily to facilitate choosing or switching to a new method, follow-up clients who were not switching methods may have been exposed to the CC materials only at their initial visit. Therefore, only follow-up clients who received counseling during a previous visit were asked about use of the CC materials.

**Table 1.** Family Planning Services Sought by Clients

Type of Family Planning Service	Total No. of Clients	No. of Clients Who Received Counseling on Day of Interview	No. of Follow-Up Clients
	**Received 1 Service**		
Obtain a method	75	75	0
Counseling	36	36	0
Follow-up/consult	109	0	109
	**Received 2 Services**		
Obtain a method and counseling	73	73	0
Obtain a method and follow-up/consult	67	67	67
Counseling and follow-up/consult	54	54	54
	**Received 3 Services**		
Obtain a method, counseling, and follow-up/consult	47	47	47
**TOTAL**	**461**	**352**	**277**^a^

a Of the 277 follow-up clients, 234 received counseling during a previous visit at the health center.

All respondents were of reproductive age, with an average age of 30 years. Slightly over half (54%) of the respondents were 30 years old or younger, 40% were 31–40 years old, and only 6% were 41–49 years old (data not shown). Nearly all respondents were married and had, on average, 3.5 children (data not shown).

#### Adherence to CC Counseling Protocol

A total of 352 women visited the health center to obtain a contraceptive method or family planning counseling. Over 80% of respondents reported that the service provider had performed the following 5 tasks (data not shown):

- Asked her whether she had a certain method in mind before coming to the center
- Explained various contraceptive methods that were available and that she could use
- Made clear what the advantages were of different contraceptive methods
- Made clear what the disadvantages were of different methods
- Discussed possible side effects of methods

Service providers were less likely to discuss the client's desired family size (62%) or to talk about the advantage of birth spacing (73%). On average, providers had performed 5.6 of these 7 tasks.

In addition, 87% of respondents reported that the provider had helped them choose a method and 70% of respondents chose a method that day. Of those who chose a method, 81% said that the provider had discussed how to manage the side effects associated with that method and 94% said the provider had explained where to obtain the method.

#### Use of CC Materials

Women who received family planning counseling (n = 352) were asked whether the provider had used CC materials. The material used most frequently was the client cue card; 73% of women reported that they had received a card during that day's visit, a near match with the 70% who reported that they had chosen a method that day. Nearly all of the women who received a client cue card expressed an intention to read the card at a later time.

About 68% recalled that the provider had used the provider cue card during the consultation and 65% noted that the provider wore the *Hayati Ahla* lab coat. Forty-four percent reported that the provider had used the wheel to identify the appropriate method.

A total of 277 women visited the health center to follow up about the method they were using at the time. Of these women, 234 had received family planning counseling at that particular center during a previous visit, 71% of whom recalled having received a client cue card during their previous visit to the health center. Nearly all of these women (98%) referred to the card once they went home, and all said that it was useful.

The Family Planning Wall Chart was noticed by 85% of all interviewed women, nearly all of whom (96%) reported that it was useful.

#### Client Satisfaction

About 4 women in every 5 were very satisfied with their visit on the day of the interview (data not shown). Satisfaction was associated with use of the CC materials, particularly the wheel, cue cards, and wearing the *Hayati Ahla* lab coat (Table 2), as well as with the provider following the CC counseling protocol (Table 3).

**Table 2.** Client Satisfaction Associated With Providers' Use of “Consult and Choose” Materials

Materials	Client Satisfaction, % (No. of Clients Reporting Provider Used Material)	Client Satisfaction, % (No. of Clients Reporting Providers Did Not Use Material)
Wheel	89.1*	72.5*
	(n = 156)	(n = 196)
Provider cue card	83.8*	71.4*
	(n = 240)	(n = 112)
*Hayati Ahla* lab coat	88.3*	63.9*
	(n = 230)	(n = 122)
Received client cue card	86.9	81.2
	(n = 221)	(n = 85)
Referred to the client cue card	89.9	86.7
	(n = 206)	(n = 15)

Client satisfaction level was defined as “very satisfied.”

* Significance comparing satisfaction between clients who recalled that their providers used materials vs. those who did not recall that their providers used the materials. *P* values ≤ .05 were considered statistically significant.

**Table 3.** Client Satisfaction Associated With Providers' Use of “Consult and Choose” Counseling Protocol

Counseling Protocol Task	Client Satisfaction, % (No. of Clients Reporting Providers Performed Task)	Client Satisfaction, % (No. of Clients Reporting Providers Did Not Perform Task)
Discussed how many children the beneficiary would like to have	87.7*	66.9*
	(n = 219)	(n = 133)
Talked to the beneficiary about advantages of birth spacing	83.0*	71.0*
	(n = 259)	(n = 93)
Asked the beneficiary whether she had a method in mind before coming to the center	82.7*	64.9*
	(n = 295)	(n = 57)
Explained available methods that the beneficiary could use	81.4*	68.9*
	(n = 307)	(n = 45)
Made clear the advantages of different methods	82.2*	67.3*
	(n = 297)	(n = 55)
Made clear the disadvantages of different methods	81.7*	71.4*
	(n = 289)	(n = 63)
Talked to the beneficiary about possible side effects of methods	82.8*	65.6*
	(n = 291)	(n = 61)
Explained how to manage side effects of the chosen method	83.6*	55.3*
	(n = 305)	(n = 47)

Client satisfaction level was defined as “very satisfied.”

* Significance comparing satisfaction between clients who recalled that their providers performed the task vs. those who did not recall that their providers performed the task. *P* values ≤ .05 were considered statistically significant.

#### Usefulness of the *Hayati Ahla* Film

We interviewed 120 clients from the 6 health centers that piloted the *Hayati Ahla* film. Nearly one-fifth of respondents had heard about the video screen in the health center from others before visiting the center, and 62% noticed a large video screen on the wall of the waiting area. Among those who noticed the screen (n = 74), 70% spent time watching the film while waiting.

The most recalled messages from the film were about (data not shown):

- Using modern methods or using contraceptive methods in general (81%)
- Spacing between pregnancies in general or spacing 3 years in particular (33%)
- Family planning counseling (14%)
- The national family planning campaign slogan of *Hayati Ahla* (14%)

As shown in Table 4, 85% of those who watched the film reported that the film influenced them. Of these, over one-third indicated that they felt more positive about using family planning and that they would now plan their families, and that they would use contraception as a direct result of viewing the film. Nearly one-fifth said they would ask a doctor about family planning. Responses were spontaneous, not prompted.

**Table 4.** Effects of the *Hayati Ahla* Video on Viewers (n = 52)

Effects Reported by Viewers	No. (%)
Will tell others about the video	52 (100.0)
Believe that such a display should be present in all health centers	52 (100.0)
Watching the video influenced the viewer	44 (84.6)
More acceptance of family planning/modern methods; less fear of modern methods	22 (45.5)
Will use contraception	16 (36.4)
Will ask doctor about family planning/modern methods	14 (31.8)
Will plan family/space 3 years between births	17 (38.6)
Will discuss family planning with spouse/others	10 (22.7)

All of the women said they would tell others about the film and 100% of the women believed that the video should be shown in all health centers.

#### Multivariate Analyses

To test whether the association between client satisfaction and use of CC materials and counseling protocol was confounded by educational attainment or age, multiple logistic regressions were conducted (Table 5). Results indicate no association between being “very satisfied” and respondents' age. Adding respondents' education to the equation increases the odds of being “very satisfied” by 30% with each increasing educational attainment category. However, this association is lost when the number of CC counseling protocol steps is inserted into the equation.

**Table 5.** Multivariate Stepwise Logistic Regression Analysis of Variables Associated With Family Planning Client Satisfaction

Variable	Odds Ratios of Being “Very Satisfied” With the Visit				
	Model 1	Model 2	Model 3	Model 4	Model 5
Age categories^a^	1.4	1.5	1.3	1.4	1.4
Educational attainment categories^b^		1.3*	1.2	1.3	1.2
Adherence to counseling protocol^c^			1.2**	–	1.1
Use of counseling materials^d^				1.7***	1.6***
No. of observations	461	461	352	352	352

Each successive model reflects the addition of a new variable in the regression analysis to test which variables remain statistically associated with client satisfaction as each new variable is added.

* *P*<.05; **P <.01; ***P <.001

a Age categories: 18–20 years, 21–30 years, 31–40 years, 41–49 years.

b Educational attainment categories: Basic or lower, secondary, intermediate diploma, university or higher.

c Number of counseling protocol steps followed according to the client's recollection.

d Number of materials used during counseling according to the client's recollection.

The odds of being “very satisfied” increases by 20% with each additional counseling protocol step performed and by 70% with each increase in the number of CC materials used.

The association between satisfaction and adherence to CC counseling protocol is lost when the number of CC materials used is incorporated into the equation, indicating that use of CC materials is a key contributor to high client satisfaction. This confounding is not surprising since the number of CC counseling steps adhered to positively correlates with the number of CC materials used (*r*^2^ = 0.5; *P*<.001, n = 352).

### Descriptive Findings From Service Statistics

In total, 14,490 referral cards were collected in MCH centers in Irbid during start-up and implementation of community-based interventions and the CC program (from June 2011 through August 2012), 59% of which were for family planning services.

About 60% of community-based referrals to health centers were for family planning services.

**Figure 1.**
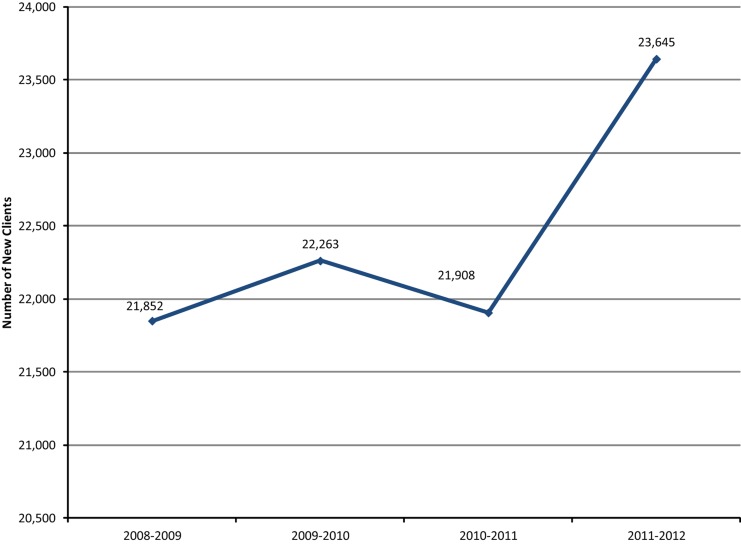
Trends in New Family Planning Clients, Irbid Governorate, August 2008–July 2012

We used the MOH's database to assess trends in new family planning users and CYP between August 2008 and July 2012 to validate the mobilization effort recorded through the referral cards. Between August 2008 and July 2011 (prior to full implementation of the Irbid Initiative), the number of new family planning clients was stagnant at around 22,000 clients per year. After launching the Irbid Initiative, the number of new clients increased to more than 23,000 during the first project year (Figure 1).

**Figure 2.**
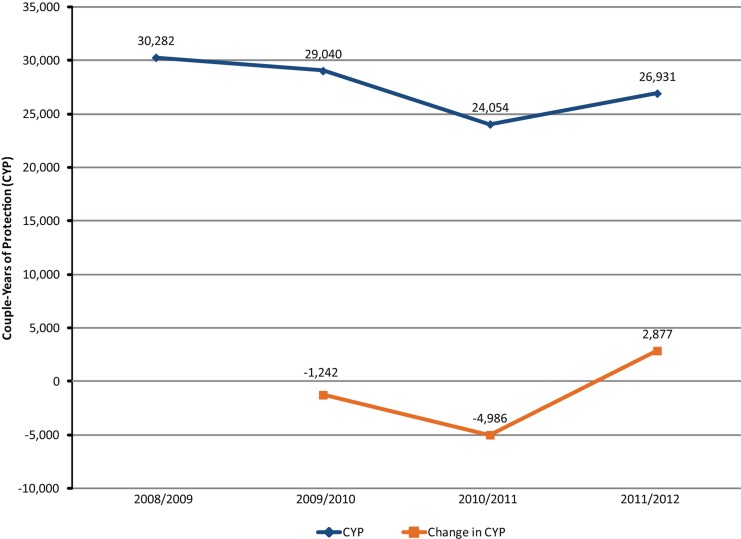
Trends in Couple-Years of Protection, Irbid Governorate, August 2008–July 2012

Long-term methods such as *Implanon*® implants and IUDs increase the CYP. Implants are not readily available in MCH centers, but the IUD is the most preferred method in Jordan.^11^ Both midwives and physicians used to insert IUDs until midwives announced in 2010 that they would no longer insert IUDs unless legislation was amended to meet their legal requirements, after which a decline in CYP was registered throughout Jordan. This decline has persisted except in Irbid, where a reversal is seen in mid-2010, which coincides approximately with implementation of the Irbid Initiative (Figure 2).

The Irbid Initiative did not attempt to address the shortage of female physicians, whom clients prefer over male providers for IUD insertion. However, the increase in CYP suggests—but does not prove—that the Irbid Initiative had positive effects in encouraging women to consider a broader range of methods given that the method mix most likely did not change from August 2008 through July 2012.

### Limitations

This evaluation was conducted in a single governorate where all family planning providers were trained to use the CC materials. Program effects on client satisfaction are very likely underestimated because even providers who did not use the CC materials as recommended had attended training sessions to build their interpersonal communication skills. It would be useful to compare the experiences of clients who visited clinics where the providers were trained in the CC approach with clinics where providers were not trained in the approach.

Asking respondents about their level of satisfaction can result in some degree of respondent acquiescence bias, which may explain, in part, the overall high levels of satisfaction with clinic visits. Since the outcome of interest is the relationship between providers' use of CC materials and client satisfaction, and not the level of client satisfaction, that potential limitation is partially mitigated.

The evaluation would have been strengthened if it had relied on third-party observation of counseling and service provision, for client recall of counseling procedures and the use of materials may have introduced a recall bias. The research team attempted to reduce recall bias by showing respondents the materials of interest and ensuring that the interview occurred within a half-hour after the beneficiary received the services.

The greater limitation, however, is that the evaluation did not follow up with the clients to assess whether use of the CC materials was associated with decreased levels of discontinuation and unmet need, the ultimate goal of the program. Although that question lies beyond the purview of this evaluation, it is an important question to address in a companion study.

Moreover, the promising trends in the CYP and new user numbers may be a result of factors external to the CC program and community-based interventions.

## DISCUSSION

Improving the quality of care of family planning services is an important goal in Jordan given the high rates of contraceptive discontinuation and unmet family planning need. It is vital that technically competent health care providers offer all family planning clients, regardless of income or educational attainment, courteous treatment and information that is accurate, understandable, and appropriate for their needs. The assumption of the CC approach is that high-quality services, which can be enhanced with appropriate training, will lead to increased clinic visits, fewer women with unmet need, and lower discontinuation rates, all of which will contribute to higher CPR and, ultimately, lower fertility rates as has been discussed in the literature.^17^-^18^

In addition to these systems-level interventions, the CC program also benefited from community-based activities that encouraged women with an unmet need for family planning to come to the health clinic, which the program tracked through referral cards. Another advantage was that the initiative was branded with the national family planning campaign logo—*Hayati Ahla—*that is displayed on all public service announcements promoting family planning, on all print materials at MCH centers, and in all community-based family planning events.

The findings demonstrate the utility of the CC approach given that client satisfaction with clinic visits was strongly and positively associated both with providers' use of the CC materials and with having a provider who, based on client perceptions, followed the CC counseling protocol. These findings add to data from other countries that showed a correlation between positive client-provider interactions with use of communication tools and enhanced client satisfaction, increased knowledge levels, and longer and more effective use of methods.^1^^,^^3^ The evaluation results also provide further support for giving clients information about their chosen method,^4^ for structured counseling,^5^-^7^ and for using screening tools and client decision aids.^8^ In addition, the family planning video shown in clinic waiting rooms amplified and extended messages about family planning, which also adds to the body of evidence about the synergistic effects of interpersonal and mediated communication.^19^-^20^

Jordan is approaching a critical stage in its development. Recent data show that Jordanians of working age (15–64 years) represent 59% of the population, and those aged 14 years or younger represent 37% of the population.^11^ The total fertility rate (TFR) decreased from 5.6 children per woman in 1990 to 3.7 children in 2002^21^; however, since then, the TFR has stagnated at 3.8 children.^11^ It is projected that Jordan will be able to realize its demographic opportunity—a state where the proportion of the working age population is at its highest and the age dependency ratio is at its minimum—only if the TFR decreases significantly by 2017 and continues its decline to 2.1 children by 2030.^21^ The implementation of the CC program at MCH centers nationally, in tandem with community-based interventions, could play a key role in attaining this goal by increasing demand for, and satisfaction from, family planning services.

The authors hope that by sharing the findings from this evaluation, the CC initiative will be replicated in Jordan and beyond to foster greater client satisfaction and long-term, effective contraceptive use among couples who desire to delay or limit childbearing. Although this initiative would likely be replicated most easily in other middle-income countries that have undergone the epidemiological transition from infectious to chronic diseases, the CC approach also could be adapted to environments with fewer resources. The CC kit included materials that have been developed and tested internationally and could readily be adapted for use in other countries, even in low-resource settings. A video or training protocol could be created to demonstrate appropriate use of these tools by providers in a culturally appropriate and practical fashion. Likewise, JHCP's close collaboration with existing community-based groups for community outreach, exemplified by AWSO^TM^, is another low-cost approach to community outreach that could be replicated. While lower-income countries may not have as many health facilities and providers who specialize in family planning service provision and counseling as in Jordan, the CC experience suggests that the appropriate and judicious use of family planning tools during clinic visits can enhance the counseling experience and lead to more satisfied family planning users.
